# Portal Vein Embolization is Associated with Reduced Liver Failure and Mortality in High-Risk Resections for Perihilar Cholangiocarcinoma

**DOI:** 10.1245/s10434-020-08258-3

**Published:** 2020-02-26

**Authors:** Pim B. Olthof, Luca Aldrighetti, Ruslan Alikhanov, Matteo Cescon, Bas Groot Koerkamp, William R. Jarnagin, Silvio Nadalin, Johann Pratschke, Moritz Schmelze, Ernesto Sparrelid, Hauke Lang, Alfredo Guglielmi, Thomas M. van Gulik, the Perihilar Cholangiocarcinoma Collaboration Group, A. Andreou, F. Bartsch, C. Benzing, S. Buettner, I. Capobianco, P. de Reuver, E. de Savornin Lohman, C. H. C. Dejong, M. Efanov, J. I. Erdmann, L. C. Franken, G. Frascaroli, M. C. Giglio, C. Gomez-Gavara, F. Heid, J. N. M. IJzermans, H. Jansson, M. A. P. Ligthart, S. K. Maithel, M. Malago, H. Z. Malik, P. Muiesan, S. W. M. Olde Damink, E. Pando, L. M. Quinn, F. Ratti, K. J. Roberts, J. Rolinger, A. Ruzzenente, E. Schadde, M. Serenari, A. Sultana, R. Troisi, S. van Laarhoven, J. L. A. van Vugt

**Affiliations:** 1 Department of Surgery, Amsterdam UMC (location AMC), University of Amsterdam, Amsterdam, The Netherlands; 2 Department of Surgery, Erasmus Medical Center, Rotterdam, The Netherlands; 3 Hepato-biliary Surgery Division, Ospedale San Raffaele-IRCCS, Milan, Italy; 4 Department of Hepato-Pancreato-Biliary Surgery, Moscow Clinical Scientific Center, Moscow, Russia; 5 Department of Medical and Surgical Sciences, S. Orsola-Malpighi Hospital, Alma Mater Studiorum – University of Bologna, Bologna, Italy; 6 Division of Hepatopancreatobiliary Surgery, Memorial Sloan-Kettering Cancer Center, New York, NY USA; 7 Department of General and Transplant Surgery, University Hospital Tübingen, Tübingen, Germany; 8 Department of General, Visceral and Transplantation Surgery, Charité University Hospital, Berlin, Germany; 9 Department of Surgery, Centre for Digestive Diseases, Karolinska University Hospital, Stockholm, Sweden; 10 Department of General, Visceral and Transplantation Surgery, University Hospital of Mainz, Mainz, Germany; 11 Department of Surgery, Unit of Hepato-Pancreato-Biliary Surgery, University of Verona Medical School, Verona, Italy

## Abstract

**Background:**

Preoperative portal vein embolization (PVE) is frequently used to improve future liver remnant volume (FLRV) and to reduce the risk of liver failure after major liver resection.

**Objective:**

This paper aimed to assess postoperative outcomes after PVE and resection for suspected perihilar cholangiocarcinoma (PHC) in an international, multicentric cohort.

**Methods:**

Patients undergoing resection for suspected PHC across 20 centers worldwide, from the year 2000, were included. Liver failure, biliary leakage, and hemorrhage were classified according to the respective International Study Group of Liver Surgery criteria. Using propensity scoring, two equal cohorts were generated using matching parameters, i.e. age, sex, American Society of Anesthesiologists classification, jaundice, type of biliary drainage, baseline FLRV, resection type, and portal vein resection.

**Results:**

A total of 1667 patients were treated for suspected PHC during the study period. In 298 patients who underwent preoperative PVE, the overall incidence of liver failure and 90-day mortality was 27% and 18%, respectively, as opposed to 14% and 12%, respectively, in patients without PVE (*p* < 0.001 and *p* = 0.005). After propensity score matching, 98 patients were enrolled in each cohort, resulting in similar baseline and operative characteristics. Liver failure was lower in the PVE group (8% vs. 36%, *p* < 0.001), as was biliary leakage (10% vs. 35%, *p* < 0.01), intra-abdominal abscesses (19% vs. 34%, *p* = 0.01), and 90-day mortality (7% vs. 18%, *p* = 0.03).

**Conclusion:**

PVE before major liver resection for PHC is associated with a lower incidence of liver failure, biliary leakage, abscess formation, and mortality. These results demonstrate the importance of PVE as an integral component in the surgical treatment of PHC.

Portal vein embolization (PVE) is considered the gold-standard procedure to enhance the future liver remnant (FLR) before major liver resection and to reduce the risk of postoperative liver failure and mortality.^1^,^2^ Since its introduction more than three decades ago, PVE has shown to induce an increase in FLR volume (FLRV) in both healthy and compromised liver parenchyma, while associated with minimal adverse events.^3^^–^5 A decrease in postoperative liver failure using PVE has frequently been reported; however, in the only prospective comparative clinical trial, undertaken by Farges et al. in 2003, PVE decreased postoperative complications only in patients with compromised liver parenchyma at increased risk of liver failure.^6^

Patients with perihilar cholangiocarcinoma (PHC) are especially at risk for liver failure due to biliary obstruction and cholestasis frequently encountered in these patients and which profoundly compromise the liver’s regenerative capacity.^7^,^8^ The vast majority of these patients require major liver resection to obtain tumor-free margins, leaving a small liver remnant that is also not able to efficiently regenerate. Therefore, biliary drainage is an essential component in the preoperative work-up of these patients in order to reduce the risk of adverse events. Liver failure and mortality rates are reported to be between 17 and 24% and 10 and 14%, respectively, and have remained high in Western series.^9^^–^13

Several studies addressed the use of PVE in patients with PHC and showed increases in liver volume; however, comparative studies demonstrating a beneficial effect of PVE on adverse events after resection are currently lacking for PHC.^14^^–^16 Therefore, this study aimed to evaluate the effect of PVE on the risk of morbidity and mortality after resection for PHC in a large multicentric Western cohort.

## Methods

All 20 participating centers included a median of 80 (25–115) consecutive resections for presumed PHC without a required fixed timespan but not preceding the year 2000. Each center included their retrospective series using a standardized and anonymized data file. PHC was defined as a suspicious biliary tumor originating at the hepatic duct confluence between the segmental bile ducts and cystic duct. For the current study, all patients who had only undergone excision of the extra hepatic bile ducts, explorative laparotomy, or liver transplantation were excluded. The need for ethical approval and individual informed consent was waived by the Institutional Medical Ethics Committee of the Amsterdam University Medical Center.

### Patient Work-Up and Management

The multicenter set-up of the current study inevitably led to differences in the work-up and management of the included patients. Therefore, the selection of patients for PVE and biliary drainage differed between centers. In general, most patients planned for large liver resections underwent preoperative, endoscopic, or transhepatic biliary drainage of at least the FLR.

### Outcome Parameters

Preoperative cholangitis was defined as fever and leukocytosis requiring (additional) biliary drainage in accordance with the definitions applied in the DROP and DRAINAGE trials dealing with preoperative biliary drainage.^17^,^18^ Major liver resection was defined as resection of at least three Couinaud liver segments. The liver remnant volume share (FLRV) was calculated by dividing the FLRV (in milliliters) by the total liver volume (in milliliters) and multiplying by 100%. R0 resection margins were defined as tumor-free margins in all reported margins in the respective pathology reports. All complications within 30 days after surgery were scored and classified according to the Dindo classification system, with grade III or higher considered as major morbidity. Liver failure, biliary leakage, and hemorrhage were scored and classified according to the respective International Study Group of Liver Surgery (ISGLS) criteria, and only grades B and C were considered as clinically relevant.^19^^–^21 Perioperative mortality was defined as death within 90 days after surgery, while overall survival was defined as the time between surgery and death, or date of last follow-up.

### Statistical Analyses

Categorical variables were reported as numbers with percentages, and tested using Chi square or Fisher’s exact tests when the expected cell count in a category was < 5. Continuous variables were displayed as median with interquartile range (IQR), and tested using Mann–Whitney *U* tests. Propensity score matching was performed using the psmatching3 plugin for SPSS using nearest-neighbor matching (1:1) with a caliper of 0.2. Matching parameters included age, sex, American Society of Anesthesiologists (ASA) classification, jaundice at presentation, biliary drainage, baseline FLRV share (before PVE), type of resection, and concomitant vascular resections. Statistical analyses were performed using SPSS (IBM Corporation, Armonk, NY, USA).

## Results

A total of 1667 patients from 20 participating centers were enrolled. Of these, the following cases were excluded: 37 un-resectable cases, 140 patients with extrahepatic bile duct resection only, and 6 patients undergoing liver transplantation. The remaining 1484 patients all underwent combined liver and biliary resection for presumed PHC (Table 1).

**Table 1:** Baseline and operative characteristics

	PVE (*n* = 298)	No PVE (*n* = 1186)	*p* Value
Age, years [median (IQR); *n* = 1484]	64 (56–71)	65 (57–72)	0.302
Male sex (*n* = 1484)	150 (50)	700 (59)	0.007
ASA classification (*n* = 1386)			0.574
I	24 (8)	127 (12)	
II	155 (54)	554 (50)	
III	104 (36)	404 (37)	
IV	4 (1)	14 (1)	
Jaundice at presentation (*n* = 1370)	233 (86)	861 (78)	0.001
Baseline bilirubin level [median (IQR); *n* = 1108]	86 (16–207)	58 (15–171)	< 0.001
Biliary drainage (*n* = 1370)			< 0.001
None	22 (8)	213 (18)	
PTBD	74 (25)	300 (25)	
EBD	138 (47)	502 (43)	
Both	61 (21)	165 (14)	
Preoperative cholangitis (*n* = 1400)	63 (21)	238 (22)	1.000
Bismuth classification (*n* = 1452)			< 0.001
Left/right duct	4 (1)	32 (3)	
I	16 (6)	41 (4)	
II	23 (8)	117 (10)	
IIIA	139 (48)	329 (28)	
IIIB	12 (4)	367 (32)	
IV	95 (33)	277 (24)	
Resection type (*n* = 1484)			< 0.001
Left hemihepatectomy	18 (6)	442 (37)	
Extended left hemihepatectomy	3 (1)	233 (20)	
Right hemihepatectomy	56 (19)	191 (16)	
Extended right hemihepatectomy	221 (74)	265 (22)	
Other	–	55 (5)	
Portal vein reconstruction (*n* = 1481)	148 (50)	327 (28)	< 0.001
Future liver remnant volume share [median (IQR)]			
Baseline (*n* = 510)	23 (19–29)	–	
After PVE (*n* = 131)	33 (27–39)	42 (31–66)	< 0.001
Preoperative bilirubin level [median (IQR); *n* = 1068]	15 (8–35)	21 (10–44)	< 0.001

Data are expressed as *n* (%) unless otherwise specified

*PVE* Portal vein embolization, *IQ R* interquartile range, *ASA* American Society of Anesthesiologists, *PTBD* percutaneous transhepatic biliary drainage, *EBD* endoscopic biliary drainage

Overall, most patients (90%) suffered from jaundice and consequently the majority underwent biliary drainage before surgery (83%), at the expense of preoperative cholangitis in 22% of patients. The majority of patients underwent either right (49%) or left (47%) liver resection, and portal vein reconstruction was performed in 32% of cases. The overall liver failure rate was 17% and 90-day mortality was 13%. 94% of patients had a pathology-confirmed diagnosis of PHC in the resection specimen, of whom 66% had tumor-free resection margins.

**Fig. 1:**
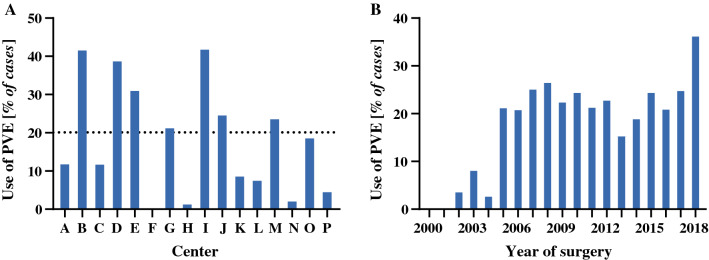
**a** Differential use of PVE across institutions with at least 15 included cases. The *dotted line* represents the use of PVE in the entire cohort. **b** Use of PVE per year in the cohort. *PVE* portal vein embolization

In this study, 298 (20%) patients underwent PVE before liver resection (Table 1). There was large variety in the use of PVE across institutions (Fig. 1a), showing a trend towards more frequent use in more recent years (Fig. 1b). The right and left liver segments were embolized in 277 (93%) and 21 (7%) patients, respectively. Segment 4 embolization was performed in 16% of all PVE procedures. In 73%, the embolic material consisted of particles in combination with coils or a plug; in 23%, glue-like materials were used; and the remaining 4% of PVE procedures were performed using a combination of the two. The rates of PVE were higher among right liver resections (38%, 277/733) compared with left liver resections (3%, 21/696), and were highest for extended right liver resections (45%, 221/486). The higher rates of biliary drainage, predominantly larger resections in right (extended) liver resections and consequently smaller remnant livers, as well as more frequent portal vein resections, indicate the higher risk of resections undertaken after PVE compared with resections without PVE. These risks are confirmed by the outcomes reported in Table 2 showing more frequent major complications, liver failure, and higher mortality in PVE patients.

**Table 2:** Postoperative outcomes

	PVE (*n* = 298)	No PVE (*n* = 1186)	*p*-Value
Pathology diagnosis (*n* = 1460)			0.830
Perihilar cholangiocarcinoma	279 (95)	1088 (93)	
Benign	6 (2)	29 (3)	
Other	8 (3)	48 (4)	
Tumor-free margin (*n* = 1417)	185 (64)	745 (66)	0.341
Morbidity Dindo grade III or higher (*n* = 1474)	177 (59)	520 (44)	< 0.001
Liver failure ISGLS grade B/C (*n* = 1472)	81 (27)	166 (14)	< 0.001
Biliary leakage ISGLS grade B/C (*n* = 1475)	59 (20)	248 (21)	0.690
Hemorrhage ISGLS grade B/C (*n* = 1214)	18 (10)	61 (6)	0.078
Intra-abdominal abscess (*n* = 1475)	50 (17)	257 (22)	0.127
90-day mortality (*n* = 1484)	53 (18)	136 (12)	0.005

Data are expressed as *n* (%)

*PVE* Portal vein embolization, *ISGLS* International Study Group of Liver Surgery

Standard left-liver resections allow for a larger liver remnant with lower operative risks, rendering PVE not often necessary in this group of patients. Assessment of the outcomes after PVE should therefore be related to the type/extent of resection. When comparing only right (extended) liver resections, the risks were more equal, with liver failure and mortality rates of 25% and 19%, respectively, in the 277 patients with PVE, compared with 23% and 16% in the 456 patients without PVE (*p* = 0.473 and *p* = 0.419); however, direct comparison of these cohorts is hampered by the wide selection of patients.

### Propensity Score Matched Cohort

In order to be able to analyze the true effects of PVE on postoperative outcomes, a propensity matched comparison was performed using only cases with complete data on all relevant parameters. After exclusion of cases with missing volume parameters, a total of 510 patients (151 with PVE and 359 without) were available for matching. Based on all parameters relevant for postoperative outcomes, two matched cohorts of 98 patients were generated (Table 3). The matched cohorts were equal in all preoperative and operative variables, including the baseline FLRV share, which increased a median of 7 percentage points after PVE. The increase in true remnant liver volume after PVE was 42% (18–59) in a median of 22 (19–29) days. The use of PVE was associated with reductions in liver failure (from 36% to 8%; 4.4-fold reduction) and biliary leakage (from 35 to 10%; 3.5-fold reduction), and a decrease in 90-day mortality (from 18 to 7%; 2.6-fold reduction).

**Table 3:** Propensity score matched comparison

	PVE (*n* = 98)	No PVE (*n* = 98)	*p*-Value
Age, years [median (IQR)]	65 (57–71)	63 (56–71)	0.606
Male sex	55 (66)	61 (62)	0.468
ASA classification			0.648
I	10 (10)	14 (14)	
II	46 (47)	46 (47)	
III	42 (43)	38 (39)	
Jaundice at presentation	74 (76)	73 (74)	1.000
Baseline bilirubin level [median (IQR)]	60 (15–213)	48 (13–135)	0.384
Biliary drainage			0.601
None	11 (11)	12 (12)	
PTBD	34 (35)	26 (27)	
EBD	29 (30)	36 (37)	
Both	24 (25)	24 (25)	
Preoperative cholangitis	28 (29)	24 (25)	0.628
Bismuth classification			0.086
Left/right duct	2 (3)	3 (3)	
I	7 (7)	3 (3)	
II	7 (7)	17 (17)	
IIIA	49 (51)	46 (47)	
IIIB	3 (3)	9 (9)	
IV	27 (28)	20 (20)	
Future liver remnant volume share [median (IQR)]			0.130
Baseline	27 (21–32)	–	
After PVE	35 (28–42)	29 (23–33)	<0.01
Preoperative bilirubin level [median (IQR)]	12 (5–27)	15 (9–38)	0.057
Resection type			0.481
Left hemihepatectomy	2 (2)	5 (5)	
Extended left hemihepatectomy	1 (1)	2 (2)	
Right hemihepatectomy	35 (36)	28 (29)	
Extended right hemihepatectomy	60 (61)	63 (64)	
Portal vein resection	18 (18)	21 (21)	0.721
Estimated blood loss [median (IQR)]	775 (500–1300)	900 (600–1996)	0.054
Morbidity Dindo grade III or higher	50 (51)	53 (54)	0.775
Liver failure ISGLS grade B/C	8 (8)	35 (36)	< 0.001
Biliary leakage ISGLS grade B/C	10 (10)	34 (35)	< 0.001
Hemorrhage ISGLS grade B/C	6 (6)	7 (7)	1.000
Intra-abdominal abscess	19 (19)	33 (34)	0.034
90-day mortality	7 (7)	18 (18)	0.031

Data are expressed as *n* (%) unless otherwise stated

*PVE* Portal vein embolization, *IQR* interquartile range, *ASA* American Society of Anesthesiologists, *PTBD* percutaneous transhepatic biliary drainage, *EBD* endoscopic biliary drainage

## Discussion

This study describes a large Western cohort of combined hepatic and biliary resections for PHC and included 1484 patients among 20 centers. Overall, 20% of patients underwent PVE before liver resection, but the use of PVE varied considerably across centers. The overall postoperative outcomes demonstrated that patients who underwent PVE were high surgical risks, showing higher rates of adverse events following resection, but any comparison is limited by selection. In a propensity score matched analysis, the effect of PVE on postoperative outcomes was assessed in two equal cohorts of 98 patients. Although preoperative parameters were similar, the incidences of liver failure, biliary leakage, intra-abdominal abscesses, and postoperative mortality were lower in the PVE group compared with patients without PVE. These outcomes in the high-risk patients who underwent PVE were all well below the rates in the overall cohort, while all rates of the matched patients without PVE stand well above those in the entire cohort.

The only prospective trial dealing with PVE showed a reduction in postoperative morbidity in patients with compromised liver parenchyma who underwent preoperative PVE; however, this trial included only patients undergoing standard right hemihepatectomy and no patients with PHC.^6^ Specifically in PHC, a study from a high-volume center in Japan reported a postoperative mortality rate of 4.5% in 132 patients who underwent PVE for an anticipated liver remnant of < 40%, while mortality was 3.7% in 136 patients who underwent resection of < 50% of liver volume without PVE.^22^ These results illustrate that PVE reduces operative risks since the former can be considered high-risk resections compared with patients with a remnant liver of 50% or higher; however, a direct comparison was not reported. The current analyses using a matched cohort of patients with and without PVE clearly demonstrates a reduction in postoperative rates of liver failure and mortality.

Although these results confirm the expected risk-reducing effects of PVE before major liver resection, PVE is only sparsely used in Western series. This is in contrast with the frequent use of PVE in Eastern series; 23 Eastern centers often report the use of PVE in the majority of patients.^23^^–^25 The largest single-center series reported use of PVE in 60% of patients and while the rates of liver failure were comparable (32%), mortality was substantially lower at only 2%.^25^ This remarkable difference in mortality has been noted across literature 23,26 and could well be due to the higher rates of PVE used in Eastern centers.^12^,^23^

The use of PVE extends the time until resection by at least 3–6 weeks in order to allow sufficient growth of the anticipated remnant liver.^5^ In the interval to surgery, these patients are at risk of developing cholangitis, which is associated with high rates of liver failure and mortality after hepatectomy.^11^,^12^,^27^,^28^ Cholangitis was not included as a matching parameter in the current analyses. In the matched comparison, the incidence of preoperative cholangitis was similar, suggesting that the increased time to surgery associated with PVE had little impact on outcomes. Furthermore, despite similar episodes of cholangitis, liver failure and mortality were reduced in patients who underwent PVE, which suggests that the protective effect of PVE overruled the negative effects of PVE.^11^,^12^ Considering the negative impact of cholangitis on outcomes, PVE should perhaps be liberally considered in this subgroup of patients, although direct evidence is lacking and will likely be difficult to obtain.

The selection of patients with PHC for PVE is a challenge since the obstructive cholestasis and accompanying biliary drainage and cholangitis are associated with loss of remnant liver function, in addition to its size alone.^12^ The most frequently used remnant volume cut-off value is 40%,^14^ but literature is equivocal and liver volume alone has insufficient predictive value for accurate patient selection for PVE.^12^,^14^,^29^ Several modalities for functional assessment of the remnant liver have been proposed to aid in the decision to perform PVE. Indocyanine green clearance tests have shown added value, over volume alone, to predict adverse outcomes, however the negative predictive value that is essential to select patients for PVE is low.^25^,^30^^–^32 In other words, a value for sufficient liver function to safely proceed without the need for PVE would benefit patient selection. Hepatobiliary scintigraphy (HBS) with technetium-labeled mebrofenin could help to achieve this goal. The usual cut-off value used in previous publications has been 2.7% min^−1^ m^−2^ based on body surface area; however, a recent report demonstrated a body surface area uncorrected remnant liver function of 8.5%/min to be safe.^29^ This relatively high cut-off value potentially leads to high rates of PVE but since complications and adverse outcomes of PVE are rare, this is likely a valuable approach to improve outcomes.^29^,^33^ Adherence to such recommendations likely reduces the relatively high liver failure rates still observed in PHC patients when adhering to the 2.7% min^−1^ m^−2^ cut-off.^29^

Although 298 patients underwent PVE in the current cohort, only 98 patients were matched, for several reasons. First, only patients with complete data, i.e. without any missing data, were included to ensure high-quality analysis. Due to the relatively low number of patients with data on liver volumes, which is an essential parameter to assess operative risks, the number of patients eligible for matching was limited. Second, patients who underwent PVE had small remnant livers, whereas a low number of patients with small remnant livers were exposed to resection without PVE due to the obvious risk of liver failure. This difference limits the ability to generate a large and well-matched cohort. Despite these limitations, the current strategy is likely the most accurate possible and the closest to a randomized trial, which will obviously not be possible to set-up because of ethical reasons. The retrospective study design is another limitation and leaves the study subject to selection bias. The time required between PVE and resection can be considered a test of time in the selection of patients with more favorable tumor biology. In addition, patients lacking an adequate hypertrophy response after PVE have likely not been subjected to surgery. Additionally, there may have been differences in patient selection for PVE as well as criteria to proceed to surgery, which could have affected the results; however, randomized studies in PHC are difficult to perform due to the rarity of the disease. The current cohort was a large Western multicenter cohort, which improves its reliability. Eastern centers were deliberately not included in the series for these analyses due to the differences in management and outcomes. Future studies should also confirm these findings in Eastern patients.^23^,^26^ Finally, some patients will have undergone PVE but no resection, however these patients were not included in this study, which could be a confounding factor. However, since approximately 37–46% 34^–^36 of patients are found to be unresectable at laparotomy regardless of PVE, including these patients would have resulted in results that would be difficult to interpret.

## Conclusion

The propensity score matched comparison in this multicenter cohort of 1484 patients showed that PVE was associated with a 4.4-fold reduction in liver failure and a 2.6-fold reduction in 90-day mortality in patients undergoing major liver resection for PHC. These outcomes in these high-risk patients after PVE are better than the outcomes in the overall cohort and show that PVE can be essential for decreasing surgical risk in these patients. Although the exact indications for PVE in patients with PHC are not clearly defined, and the use of PVE varies widely across centers, a liberal approach to the application of PVE in patients with future livers remnant < 40% is likely to improve postoperative outcomes.
